# High Genetic Diversity and Structured Populations of the Oriental Fruit Moth in Its Range of Origin

**DOI:** 10.1371/journal.pone.0078476

**Published:** 2013-11-04

**Authors:** Yan Zheng, Xiong Peng, Gaoming Liu, Hongyan Pan, Silvia Dorn, Maohua Chen

**Affiliations:** 1 College of Plant Protection, Northwest A&F University, Yangling, China; 2 ETH Zurich, Applied Entomology, Zurich, Switzerland; 3 Key Laboratory of Crop Pest Integrated Pest Management on the Loess Plateau of Ministry of Agriculture, Yangling, China; University of California, Berkeley, United States of America

## Abstract

The oriental fruit moth *Grapholita ( = Cydia) molesta* is a key fruit pest globally. Despite its economic importance, little is known about its population genetics in its putative native range that includes China. We used five polymorphic microsatellite loci and two mitochondrial gene sequences to characterize the population genetic diversity and genetic structure of *G. molesta* from nine sublocations in three regions of a major fruit growing area of China. Larval samples were collected throughout the season from peach, and in late season, after host switch by the moth to pome fruit, also from apple and pear. We found high numbers of microsatellite alleles and mitochondrial DNA haplotypes in all regions, together with a high number of private alleles and of haplotypes at all sublocations, providing strong evidence that the sampled area belongs to the origin of this species. Samples collected from peach at all sublocations were geographically structured, and a significant albeit weak pattern of isolation-by-distance was found among populations, likely reflecting the low flight capacity of this moth. Interestingly, populations sampled from apple and pear in the late season showed a structure differing from that of populations sampled from peach throughout the season, indicating a selective host switch of a certain part of the population only. The recently detected various olfactory genotypes in *G. molesta* may underly this selective host switch. These genetic data yield, for the first time, an understanding of population dynamics of *G. molesta* in its native range, and of a selective host switch from peach to pome fruit, which may have a broad applicability to other global fruit production areas for designing suitable pest management strategies.

## Introduction

In insect field populations, genetic diversity and genetic structure can be affected by various factors, such as range of origin as opposed to invaded regions, geographical distance and host plant species [1]–[3]. Some studies have documented that genetic diversity and structure are associated with the dispersal ability of the insect concerned [4]–[6]. Generally, populations in their native range show a particularly high genetic diversity, whilst populations in invaded regions usually show reduced genetic diversity [7], [8].

An insect species of global importance is the oriental fruit moth, *Grapholita* (* = Cydia*) *molesta* Busck (Lepidoptera: Tortricidae), a key pest of rosaceae fruit trees wordwide [9]. Its putative native range includes China, where it is thought to have co-evolved with its primary host peach [9], [10], but little is known yet about its population genetics in this area. This moth completes three to seven generations per year depending on latitude and elevation [11], [12]. Remarkably, it makes a host switch from peach to pome fruit in the late part of the season, particularly after peach harvest, in several areas of the world including China [11], [12], Italy [13], [14], Southern Switzerland [15] and USA [16]. The changing abiotic environment in the late season with decreasing daylight period experienced in the larval stage promotes flight performance in this species [17]. In addition, changing compositions of volatile blends emitted by its primary host peach and its secondary hosts apple and pear further contribute to this host switch, as volatiles from the pome fruit apple and pear become particularly attractive to female moths in the late season [18]–[21]. Despite the importance of the seasonal dynamics given by this host switch, consequences on patterns of population structure in the oriental fruit moth are yet unknown.

Dispersal of *G. molesta* is considered limited based on estimation of its flight range [22], [23], though a proportion of individuals within a population, in particular females, have the ability to disperse between orchards [17]. It is expected that *G. molesta* individuals have an ecological advantage when they remain and oviposit within the orchard where they emerge, unless environmental change triggers displacement of some individuals [4]. Whereas no detailed information is available on population structure in the native range, structured populations of *G. molesta* have been reported twice from invaded countries. In South Africa, *G. molesta* populations collected from closely situated orchards (<1 km) could be distinguished [4], and significant intra-regional structure among five populations from eight sampling sites were reported for this species in Italy [24]. A recent paper analyzed the invasion routes of *G. molesta* on different continents, and documented that populations of this species were geographically structured on a continental scale [10]. Three populations from China were used in that above work to illustrate Asian populations, however, they were not representative of a broad geographic sampling range or of a main fruit growing region in this country.

Population genetic studies can reveal the micro-evolution and ecological adaption strategies of an insect pest in agroecosystems [3], and thereby greatly advance our understanding of its genetic diversity and genetic structure. Such knowledge is important for the design and optimization of sustainable pest management strategies [4], [5], [24]. Our objective was to investigate the genetic diversity of *G. molesta* in its putative range of origin and its genetic structure in a key fruit growing area of China, as well as the consequence of seasonal dynamics with host switch from primary to secondary hosts. We analyzed samples collected from peach throughout the season and from pome fruit after host switch in the late season, using a combination of microsatellite data and mitochondrial gene sequences. We hypothesized that populations will show (a) a number of typical characteristics reflecting a native species, (b) a genetic structure between different regions and possibly also between sublocations within these regions, reflecting the limited flight capacity of this moth species, and (c) little genetic structure across the season irrespective of the host infested.

## Materials and Methods

### Ethics Statement

No specific permissions were required for the described field studies for this wide spread agriculture pest. We confirm that the locations were not privately owned or protected in any way. The field studies did not involve endangered or protected species.

### Insect sampling

**Figure 1:**
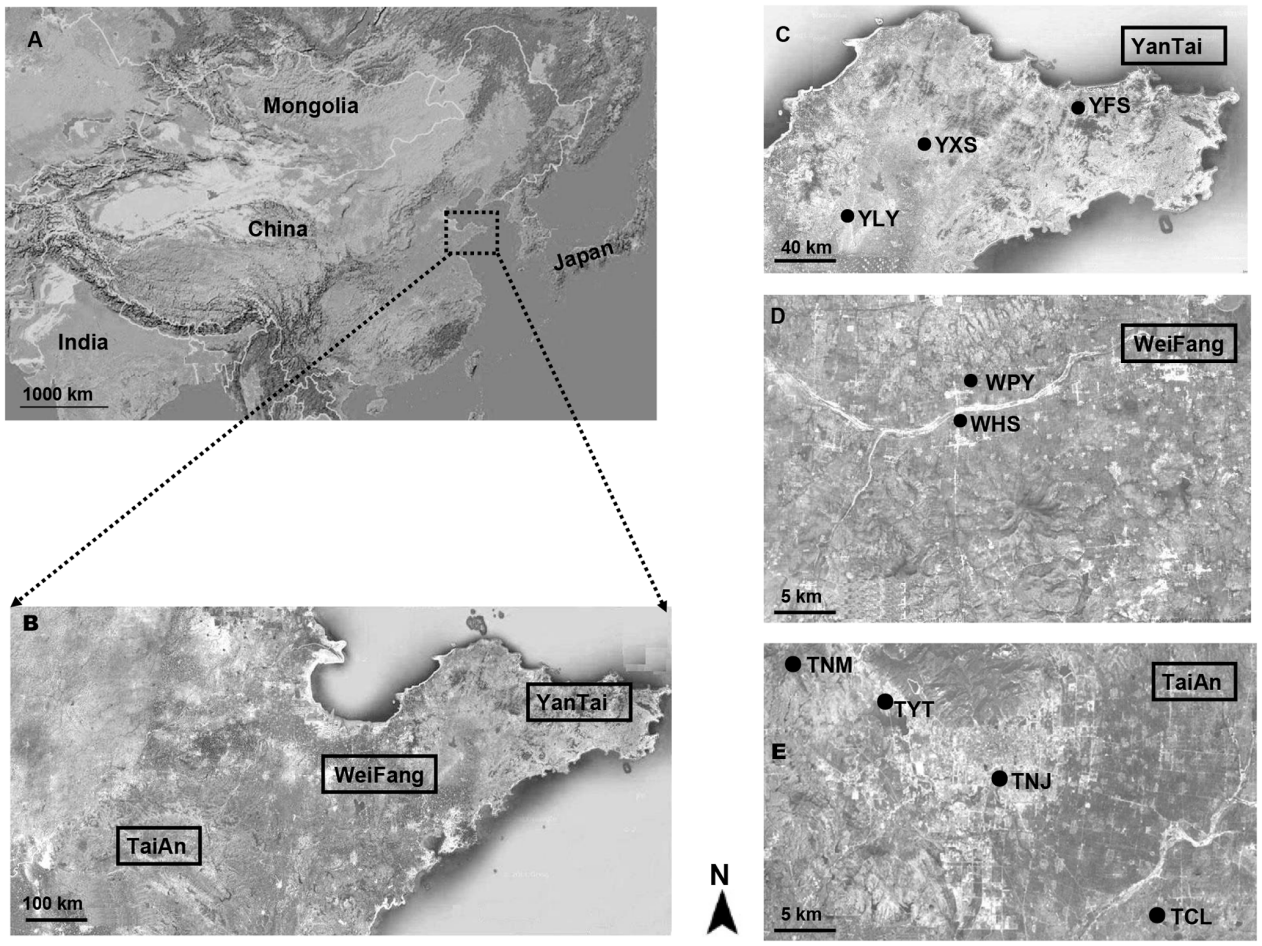
Sampling regions and sublocations of *G. molesta* in China. The codes for sublocations are explained in Table 1. (A) map of China (*dashed box* indicates the sampling regions); (B) enlarged map of sampling regions (*real line box* indicates three sampling regions); sampling sublocations in (C) YanTai region; (D) WeiFang region; (E) TaiAn region.

Oriental fruit moth larval samples were collected in 2011 from a major Chinese fruit growing area, where this species is found on peach until peach harvest, which is only in exceptional cases after July, and on the pome fruit apple and pear, where it is found nearly exclusively in the late season, i.e. in August. Samples from 9 sublocatons in 3 regions were used (Fig. 1), and all orchards sampled (>5 ha) consisted of trees of the same host plant species. To test for potential seasonal variations, samples were taken at different times from the same orchard wherever possible (Table 1). The distance between sampled fruit trees was at least 5 m, and only one larva per tree was used to minimize sibling collection. At least 50 2^nd^ to 3^rd^ instar larvae were collected per population (sublocation, host species and time; Table 1), and preserved in 10 ml Falcon tubes filled with ethanol.

**Table 1:** Sampling of *Grapholita molesta* in three Chinese regions on different hosts at different times.

Region	Sublocation	*SLC*	Coordinates	*D*	Host	*PC*	*N*
TaiAn	NingJia	TNJ	117°09.386′, 36°10.244′	17Jun.	Peach	6TNJ-Ph	24
				19Jul.	Peach	7TNJ-Ph	18
				21Jul.	Peach	7TNM-Ph	26
	NanMa	TNM	117°00.521′, 36°15.543′	22Aug.	Peach	8TNM-Ph	26
	YinTao	TYT	117°03.372′, 36°12.511′	21Jun.	Peach	6TYT-Ph	26
				23Jul.	Peach	7TYT-Ph	24
	CuLai	TCL	117°05.951′, 36°12.473′	25Jul.	Peach	7TCL-Ph	24
				23Aug.	Apple	8TCL-Ap	22
YanTai	FuShan	YFS	121°16.441′, 37°29.162′	08Jul.	Peach	7YFS-Ph	23
			121°16.394′, 37°28.811′	24Aug.	Pear	8YFS-Pr	24
	XiSanLi	YXS	120°48.071′, 37°18.388′	24Aug.	Peach	8YXS-Ph	24
			121°48.673′, 37°18.319′	24Aug.	Apple	8YXS-Ap	22
			121°48.673′, 37°18.319′	24Aug.	Apple	8YXS-Ap	22
	LaiYang	YLY	120°43.974′, 36°55.372′	24Aug.	Pear	8YLY-Pr	24
WeiFang	HongSha	WHS	118°56.300′, 36°20.284′	22Jun.	Peach	6WHS-Ph	24
				16Jul.	Peach	7WHS-Ph	24
				19Aug.	Peach	8WHS-Ph	24
	PingYuan	WPY	118°56.606′, 36°21.408′	19Aug.	Peach	8-WPY-Ph	18

Information including region, sublocation code (*SLC*), sublocation, latitude and longitude, collection date (*D*), host, population code (*PC*) and number of individuals (*N*). Sublocation codes of the sampling region are indicated with a same initial letter. The population code (*PC*) shows: samples collected in the same month (June, July or August) indicated by the same number (6, 7 or 8, resp.), samples from the same sublocation with the same three capital letters (sub location code) in the middle with the initial letter referring to the region, and samples from the same host plant with the same two letters at the end (peach (Ph), pear (Pr) or apple (Ap)).

### DNA extraction

Genomic DNA was extracted from 8–10 mg of larval material using the DNeasy Tissue Kit (Qiagen, Hilden, Germany). Extraction was performed according to the bench protocol for animal tissues. DNA was eluted in TE buffer and stored at −20°C.

### Microsatellite amplification and genotyping

Ten previously reported microsatellite markers [24] were used in all the samples. Data of five loci (*Gm01*, *Gm03*, *Gm04*, *Gm05* and *Gm07*) had to be rejected either because the loci were monomorphic (*Gm01*, *Gm04*, and *Gm05*), or because of the presence of stutter peaks and/or imperfect repeats (*Gm03* and *Gm07*) in genotyping results of many samples. Five loci (*Gm02*, *Gm06*, *Gm08*, *Gm09* and *Gm10*) that were highly polymorphic were selected for further analysis. The forward primer of each pair was labeled at the 5′ end with FAM fluorescent dye [25]. Amplifications were carried out in a total volume of 25 µl, containing 1×PCR amplification buffer (Takara, Dalian, China), 2 µM MgCl_2_, 0.2 µM of each dNTP (Takara, Dalian, China), 0.5 µl of each forward primer (2 µM), 2 µl of each reverse primer (2 µM), 2 µl M13 primer (2 µM), 1.0 U Taq polymerase (Takara, Dalian, China), and 2 µl genomic DNA (10–30 ng/µl). Amplification comprised a 10 minutes initial denaturation at 95°C, followed by 35 cycles of 30 seconds at 95°C, 45 seconds annealing at a primer-specific temperature, and 45 seconds at 72°C, then 10 cycles of 30 seconds at 95°C, 45 seconds at 53°C and 45 seconds at 72°C, and a final step 10 minutes at 72°C. To examine the length and genotype of the amplified PCR products, an ABI3730XL automated DNA sequencer (Applied Biosystems, Foster City, CA, USA) and GENESCAN version 4.0 (Applied Biosystems, Foster City, CA, USA) were used.

### Mitochondrial DNA amplification

The 740 bp mitochondrial cytochrome oxidase subunit I (COI) was amplified with the primer pairs C1-J-2183 (5′-CAACATTTATTTTGATTTTTTGG -3′) and TL2-N-3014 (5′-TCCAATGCACTAATCTGCCATATTA -3′) [26], while 700 bp cytochrome oxidase subunit II (COII) was amplified using the primer pairs TL2-J-3037 (5′-ATGGCAGATTATATGTAATGG-3′) and TK-N-3785 (5′-GTTTAAGAGACCAGTACTTG-3′) [26]. Amplifications were carried out in a total volume of 25 µl, containing 1×PCR amplification buffer (Takara, Dalian, China), 2 µM MgCl_2_, 0.2 µM of each dNTP (Takara, Dalian, China), 3.0 µl of each oligonucleotide primer (2 µM), 2 µl genomic DNA (10–30 ng/µl). The thermal profile used a 10 minutes initial denaturation at 95°C, followed by 35 cycles of 30 seconds at 95°C, 45 seconds annealing at a primer-specific temperature (48°C for COI and 57°C for COII), 45 seconds at 72°C, and a final step of 15 minutes at 72°C. After verification via gel electrophoresis, the PCR templates were purified and then sequenced in both directions using the same primer pairs on an ABI3730XL automated DNA sequencer (Applied Biosystems, Foster City, CA, USA).

### Data analysis

We used MICRO-CHECKER version 2.2.3 [27] to check the data for null alleles, stutter-errors and large allele drop-out. Null allele frequencies at each locus were calculated using the R PACKAGE GENELAND 3.1.4 [28], [29]. GENEPOP version 4.0.1 [30] was used to calculate allele frequencies, number of alleles per locus, the observed (*H_o_*) and expected heterozygosity (*H_e_*), inbreeding coefficient (*F_IS_*), the genotypic linkage disequilibrium, deviations from Hardy-Weinberg equilibrium, and the Hardy-Weinberg equilibrium *P* values. ADZE (Allelic Diversity Analyzer) version 1.0 [31] was utilized for calculating the mean allelic richness per locus for all the sampling populations with a rarefaction method that adjusted the observed number of alleles to a common size for each population sample.

Population structure from microsatellite data was analyzed using STRUCTURE version 2.3.3 [32] based on Bayesian clustering approach. We used the admixture ancestry model and the correlated allele frequency model. The correct number of clusters (*K*) was determined by calculating the probability of the data given a certain prior value of *K*. Markov chain Monte Carlo (MCMC) was carried out using 20 replicate runs of 1 000 000 iterations following a burn-in period of 50 000 iterations for each *K*-value from 1 to 10. The most likely value of *K* was estimated according to Evanno et al. (2005) [33]. The Nei's standard genetic distances among populations were utilized to construct Neighbor-joining (NJ) tree using PHYLIP version 3.6a [34]. The tree was visualized using TREEVIEW version 2.0 software [35]. In addition, we determined private allele estimates using HP-RARE [36].

We used ARLEQUIN version 3.5.1.2 [37] to estimate the molecular variance (AMOVA) between the groups of populations, along with the pair fixation indices (*F_ST_*), and significance levels were assessed (*P*-values) using 10,000 permutations of diploid multilocus genotypes between samples [38]. A two-tailed *t* test at the significant level 0.05 was used to test whether differentiation between pome fruit populations was less than differences between pome fruit and peach populations; pairwise *F_ST_* values between pome fruit populations were compared to pairwise *F_ST_* values difference between pome fruit and peach populations in the test.

For AMOVA, samples were grouped according to three models: (A) ‘comparison of variance among peach populations from different regions’. In this model, 13 populations from peach were divided into three groups by geographic regions: (i) TaiAn (6TNJ-Ph, 6TYT-Ph, 6TNJ-Ph, 7TNM-Ph, 7TYT-Ph, 7TCL-Ph and 8TNM-Ph); (ii) WeiFang (6WHS-Ph, 7WHS-Ph, 8WHS-Ph and 8WPY-Ph); (iii) YanTai (7YFS-Ph and 8YXS-Ph). (B) ‘comparison of variance among pome fruit populations from different regions’. In this model, genetic differentiation of four pome fruit populations from different regions were separated into two groups according to geographic regions: (i) TaiAn (8TCL-Ap); (ii) YanTai (8YXS-Ap, 8YFS-Pr and 8YLY-Pr). (C) ‘comparison of variance among populations from peach and pome fruits. In this model, all 17 populations were divided into two groups by the types of host plants: (i) peach (6TNJ-Ph, 6TYT-Ph, TNJ-Ph, 7TNM-Ph, 7TYT-Ph, 7TCL-Ph, 8TNM-Ph, 6WHS-Ph, 7WHS-Ph, 8WHS-Ph, 8WPY-Ph, 7YFS-Ph, and 8YXS-Ph); (ii) pome fruit (8TCL-Ap, 8YXS-Ap, 8YFS-Pr and 8YLY-Pr).

In order to test for isolation by distance (IBD), the matrices of genetic distance *F_ST_*/(1- *F_ST_*) of microsatellite data and the geographic distance (lnKm) were compared using the Mantel test with10,000 permutations [39]. This analysis was performed using the ZT-software [40].

The obtained mitochondrial DNA sequences were aligned using CLUSTAL_X version1.8 [41]. All population genetic parameters including number of haplotypes (*Nh*), nucleotide diversity (π) and haplotype diversity (*Hd*) were calculated using the program DnaSP version 5.0 [42].

To study the genetic relationships among haplotypes, we used the program NETWORK 4.6.1 [43] to construct the Median-joining networks of mtDNA haplotypes basing on statistical parsimony, and Program MEGA 5.0 [44] was used to reconstruct the NJ tree under the p-distances simple assumptions.

## Results

### Microsatellite marker and mitochondrial DNA

We genotyped 399 individuals at five microsatellite loci. Population statistics for the 399 analyzed individuals of 17 investigated populations are given in Table 2. The mean number of alleles per locus ranged from 7.0 to 16.4, reflecting relative higher numbers of alleles in comparison with the invasive populations of the pest [24]. No significant linkage disequilibrium (*P*<0.05) was found of all possible combinations after Bonferroni correction, which suggests that the five loci represented independent information across all the samples. The frequency of null alleles ranged from 0.13 to 0.28, which are similar values to previously reported ones from this tortricid pest [3], [24] and are typical for lepidopterans [5], [24], [45], [46].

**Table 2:** Population statistics for *G. molesta* investigated using five microsatellites.

Population	*N*	*N_A_*	*P_A_*	*r*	*H_o_*	*H_e_*	*F_IS_*	HW-*P*
6TNJ-Ph	24	8.8	2.0	8.1	0.466	0.765	0.396	0.060
6TYT-Ph	18	7.8	2.0	7.8	0.272	0.752	0.648	**0.000**
7TNJ-Ph	26	9.8	2.4	8.8	0.354	0.776	0.571	**0.000**
7TNM-Ph	26	12.4	5.2	10.5	0.441	0.751	0.577	**0.020**
7TYT-Ph	26	11.2	3.8	9.7	0.625	0.859	0.409	**0.030**
7TCL-Ph	24	9.6	2.4	8.9	0.331	0.762	0.226	0.100
8TNM-Ph	24	12.6	4.8	11.3	0.618	0.789	0.275	0.190
8TCL-Ap	22	16.4	8.8	14.9	0.530	0.894	0.436	**0.000**
6WHS-Ph	23	8.4	2.2	7.9	0.348	0.689	0.501	0.070
7WHS-Ph	24	6.4	1.4	6.2	0.469	0.531	0.316	**0.030**
8WHS-Ph	24	8.6	1.8	8.0	0.508	0.754	0.293	0.170
8WPY-Ph	24	7.8	2.6	7.1	0.389	0.683	0.387	0.090
7YFS-Ph	24	10.2	3.0	9.4	0.525	0.810	0.358	0.120
8YXS-Ap	24	15.8	7.8	13.8	0.535	0.835	0.266	**0.010**
8YXS-Ph	24	7.0	1.6	6.8	0.560	0.658	0.334	0.140
8YFS-Pr	24	10.8	4.2	10.0	0.576	0.807	0.307	0.100
8YLY-Pr	18	13.2	6.0	13.3	0.422	0.851	0.510	**0.000**

*N*, number of moths successfully genotyped; *N_A_*, mean number of alleles per locus; *P_A_*, number of private alleles; *r*, allelic richness; *H_o_*, observed heterozygosity; *H_e_*, expected heterozygosity; *F_IS_*, multilocus estimate of inbreeding coefficient; HW-*P*, *P*-value for Hardy-Weinberg equilibrium (significant departures from HW equilibrium are given in bold, *P*<0.05); *H_e_*, *H_o_*, *F_IS_*, *N_A_*, *P_A_* and HW-*P* are all indicated by mean values over five loci. The significances were tested for multi comparisons using the Bonferroni method.

In all the 399 individuals analyzed, we obtained 385 COI (740 bp) and 396 COII (700 bp) gene sequences, and observed 34 haplotypes for the COI gene (GenBank accession numbers are KF552033 to KF552066) and 38 haplotypes for the COII gene (GenBank accession numbers are KF551995 to KF552032).

### Genetic variation and genetic diversity

#### Microsatellite analysis

In the analyzed *G. molesta* populations sampled in China (Table 2), a total of 126 alleles were found across the five genotyped loci. The mean number of alleles per locus ranged from 6.4 to16.4. We found a high number of private alleles (between 1.4 and 8.8 per population) as well as high allele richness (between 6.2 and 14.9). The mean value of *H_e_* was between 0.66 and 0.89, that of *H_o_* between 0.27 and 0.63. Eight of the 17 populations revealed significant departures from Hardy-Weinberg equilibrium, together with high positive mean values of *F_IS_* ranging from 0.23 to 0.65, indicating the existence of heterozygote deficiencies (Table 2).

#### mtDNA gene analysis

Five of the observed 34 *G. molesta* COI gene haplotypes were shared between populations, 29 were private. Sixteen of 38 COII gene haplotypes were shared between populations, 22 were private. The COII and COI alignment had 21 (3%) and 28 (4%) variable characters, respectively, and no indels were observed in either of the two gene regions. As is common for an insect mitochondrial gene, the base composition of each of the two genes was biased toward As and Ts (76.4% of COI and 72.7% of COII). The COI gene covered 28 variable sites, 18 of which were parsimony informative, whilst 13 of the 21 polymorphic sites in COII gene were parsimony informative.

The haplotype diversity (*Hd*) ranged from 0.63 to 0.95 for COII, and from 0.34 to 0.87 for COI (Table 3). The number of haplotypes was remarkably high. The average number of haplotypes (*Nh*) of COI over all populations was 5.06, ranging from 3 to 10, and for COII, the mean number of haplotypes was 8.12, ranging from 5 to 14. The average nucleotide diversity (π) of COI was 0.14%, ranging from 0.11% to 0.24%, for COII the mean nucleotide diversities (π) was 0.26%, ranging from 0.13% to 0.38% (Table 3).

**Table 3:** Genetic diversity of 17 *G. molesta* populations as revealed by two mitochondrial genes.

mtDNA	*PC*	*N*	*Nh*	*π* (%)	*Hd*
COI	6TNJ-Ph	24	4	0. 12	0.659
	6TYT-Ph	18	5	0. 21	0.621
	7TNJ-Ph	26	3	0. 06	0.335
	7TNM-Ph	26	5	0. 12	0.508
	7TYT-Ph	26	6	0. 14	0.668
	7TCL-Ph	24	4	0. 11	0.620
	8TNM-Ph	23	5	0. 12	0.628
	8TCL-Ap	15	4	0. 15	0.695
	6WHS-Ph	23	6	0. 14	0.739
	7WHS-Ph	24	4	0. 11	0.656
	8WHS-Ph	23	3	0. 10	0.625
	8WPY-Ph	24	10	0. 24	0.866
	7YFS-Ph	24	6	0. 14	0.754
	8YXS-Ap	19	5	0. 13	0.708
	8YXS-Ph	24	4	0. 12	0.663
	8YFS-Pr	24	7	0. 14	0.739
	8YLY-Pr	18	5	0. 16	0.680
COII	6TNJ-Ph	23	9	0. 30	0.880
	6TYT-Ph	18	7	0. 25	0.771
	7TNJ-Ph	26	8	0. 19	0.803
	7TNM-Ph	26	10	0. 25	0.865
	7TYT-Ph	26	10	0. 32	0.809
	7TCL-Ph	24	6	0. 13	0.630
	8TNM-Ph	24	7	0. 20	0.833
	8TCL-Ap	22	8	0. 22	0.827
	6WHS-Ph	23	14	0. 31	0.929
	7WHS-Ph	24	7	0. 22	0.804
	8WHS-Ph	24	7	0. 23	0.801
	8WPY-Ph	24	14	0. 38	0.946
	7YFS-Ph	24	5	0. 21	0.746
	8YXS-Ap	22	5	0. 27	0.792
	8YXS-Ph	24	8	0. 30	0.855
	8YFS-Pr	24	7	0. 30	0.819
	8YLY-Pr	18	6	0. 24	0.824

*PC*, population code; *N*, sample size; *Nh*, number of haplotypes; *π*, nucleotide diversity; *Hd*, haplotype diversity.

### Genetic structure

#### Microsatellite cluster analysis

**Figure 2:**
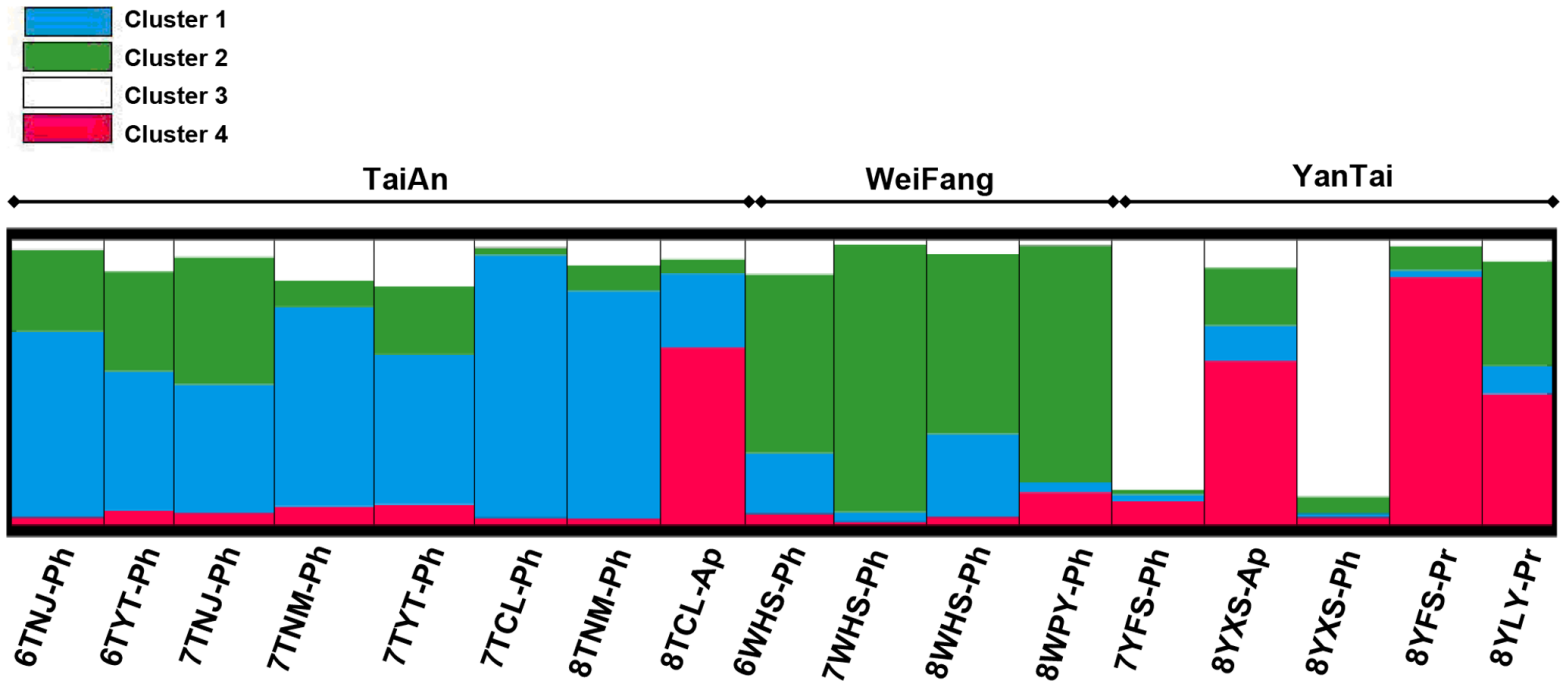
Bayesian clustering analysis using STRUCTURE indicating the presence of four clusters. Proportion of membership coefficient for 17 *G. molesta* populations falling into the four clusters is depicted by four different colors, respectively. The codes for populations are explained in Table 1.

The most likely value of *K* was 4 [46] indicating that the 17 populations included in this study can be assigned to four clusters, which are hereafter referred to as *cluster 1*, *cluster 2*, *cluster 3* and *cluster 4*. The proportions of each population that contributed to each of the four clusters are shown in Fig. 2. *Cluster 1* mainly consisted of individuals from the host plant peach in the TaiAn region (6TNJ-Ph, 0.652, implying that 65.2% of individuals from 6TNJ-Ph contributed to this cluster; 6TYT-Ph, 0.489; 7TNJ-Ph, 0.451; 7TNM-Ph, 0.701; 7TYT-Ph, 0.527; 7TCL-Ph, 0.925 and 8TNM-Ph, 0.800). *Cluster 2* comprised all four populations sampled from peach at two sublocations in the WeiFang region (6WHS-Ph, 0.629; 7WHS-Ph, 0.939; 8WHS-Ph, 0.632 and 8WPY-Ph, 0.832). *Cluster 3* was mainly made up of two populations sampled from peach in the YanTai region (7YFS-Ph, 0.876 and 8YXS-Ph, 0.90). *Cluster 4* primarily contained individuals from the populations sampled in the late season from apple and pear in several regions (8TCL-Ap, 0.623; 8YXS-Ap, 0.576; 8YFS-Pr, 0.870 and 8YLY-Pr, 0.460).

#### Neighbor-joining tree derived from microsatellite allele frequencies

**Figure 3:**
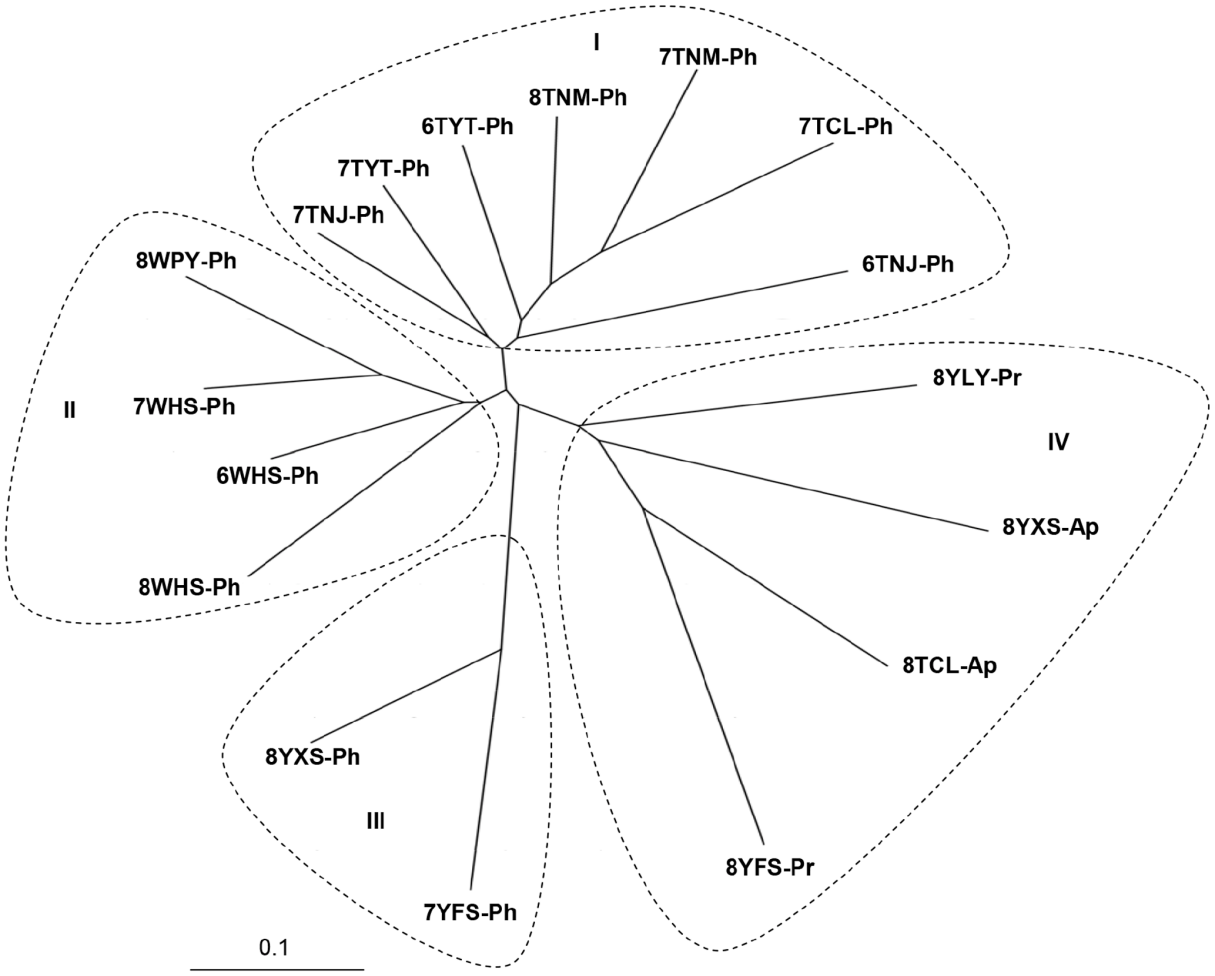
Unrooted NJ tree based on Nei's genetic distances at five microsatellite loci. Seventeen *G. molesta* populations were assigned to four clades indicated by I, II, III, IV in correspondence to clusters 1–4 of the Bayesian clustering analysis. I, samples from peach in TaiAn; II, samples from peach in WeiFang; III, samples from peach in YanTai; IV, samples from pear and apple in TaiAn, WeiFang or YanTai. The codes for sublocations are explained in Table 1.

To assess whether the four clusters obtained by Bayesian clustering analysis using Structure were genetically distinct, we further analysed the relationship among populations using NJ tree [47] based on Nei's genetic distance (Fig. 3). The NJ tree resulted in four major clades which were consistant with the Bayesian clustering results of the microsatellite data. All seven populations (6TNJ-Ph, 6TYT-Ph, 7TNJ-Ph, 7TNM-Ph, 7TYT-Ph, 7TCL-Ph, and 8TNM-Ph) collected from peach in TaiAn made up one clade. Similarly, all four populations (6WHS-Ph, 7WHS-Ph, 8WHS-Ph, and 8WPY-Ph) sampled from peach in WeiFang were found in the same clade, and the two populations (7YFS-Ph and 8YXS-Ph) from peach in YanTai formed a clade. Populations (8TCL-Ap, 8YXS-Ap, 8YFS-Pr and 8YLY-Pr) sampled in the late season from the pome fruit apple and pear of various regions constitute a single clade, which revealed the same genetic structure as the afore mentioned Bayesian clustering analysis.

#### Pairwise *F_ST_* analysis from microsatellite loci

Pairwise *F_ST_* analysis was calculated for each pair of populations over five microsatellite loci with values ranging from 0.021 to 0.255. The significance of pairwise *F_ST_* was determined with a Markov chain analysis. The results showed significant differentiation between all pairs of populations with the exception of one pair (6TYT-Ph and 7TYT-Ph) (Table 4). The two-tailed *t* test results showed that the differentiation among pome fruit populations (mean pairwise *F_ST_* = 0.022±0.0002) was less than the differentiation between pome fruit and peach populations (mean pairwise *F_ST_* = 0.050±0.002) with a *P* value of 0.010 (*t* = 1.967, *df* = 325).

**Table 4:** The range of values averaging five microsatellite loci between G. molesta populations and the significance of population differentiation estimated by *F_ST_* values with *P<0.05 and **P<0.01.

	6TNJ-Ph	6TYT-Ph	7TNJ-Ph	7TNM-Ph	7TYT-Ph	7TCL-Ph	8TNM-Ph	8TCL-Ap	6WHS-Ph	7WHS-Ph	8WHS-Ph	8WPY-Ph	7YFS-Ph	8YXS-Ap	8YXS-Ph	8YFS-Pr
6TNJ-Ph																
6TYT-Ph	0.094**															
7TNJ-Ph	0.081**	0.074**														
7TNM-Ph	0.118**	0.081**	0.101**													
7TYT-Ph	0.098**	0.021	0.081**	0.077**												
7TCL-Ph	0.127**	0.094**	0.103**	0.079**	0.117**											
8TNM-Ph	0.069**	0.054**	0.073**	0.059**	0.049**	0.040*										
8TCL-Ap	0.080**	0.076**	0.081**	0.100**	0.080**	0.067**	0.042*									
6WHS-Ph	0.095**	0.088**	0.073**	0.145**	0.122**	0.126**	0.098**	0.113**								
7WHS-Ph	0.167**	0.153**	0.112**	0.232**	0.184**	0.230**	0.197**	0.210**	0.057**							
8WHS-Ph	0.085**	0.112**	0.055**	0.131**	0.115**	0.103**	0.086**	0.104**	0.054**	0.102**						
8WPY-Ph	0.142**	0.127**	0.094**	0.169**	0.141**	0.145**	0.117**	0.113**	0.072**	0.116**	0.093**					
7YFS-Ph	0.161**	0.120**	0.126**	0.135**	0.106**	0.123**	0.079**	0.094**	0.160**	0.255**	0.145**	0.170**				
8YXS-Ap	0.092**	0.055**	0.067**	0.095**	0.058**	0.123**	0.073**	0.063**	0.107**	0.161**	0.112**	0.120**	0.095**			
8YXS-Ph	0.189**	0.091**	0.134**	0.159**	0.111**	0.208**	0.167**	0.169**	0.151**	0.170**	0.175**	0.187**	0.121**	0.091**		
8YFS-Pr	0.115**	0.107**	0.119**	0.136**	0.094**	0.134**	0.088**	0.050**	0.164**	0.251**	0.139**	0.161**	0.147**	0.088**	0.209**	
8YLY-Pr	0.062**	0.064**	0.055*	0.104**	0.075**	0.088**	0.055**	0.044*	0.064**	0.122**	0.051*	0.075**	0.105**	0.040*	0.133**	0.071**

#### Isolation by distance

The Mantel test provided an *r* value of 0.363 (*P* = 0.005) based on all 13 populations from peach, indicating that there was a significant isolation-by-distance effect present among different geographic populations from this host. In contrast, when the four populations from the pome fruit hosts were added, this correlation between genetic distances and geographic distances was no longer significant (*r* = 0.086, *P* = 0.253).

#### Haplotype network of the combined mtDNA

**Figure 4:**
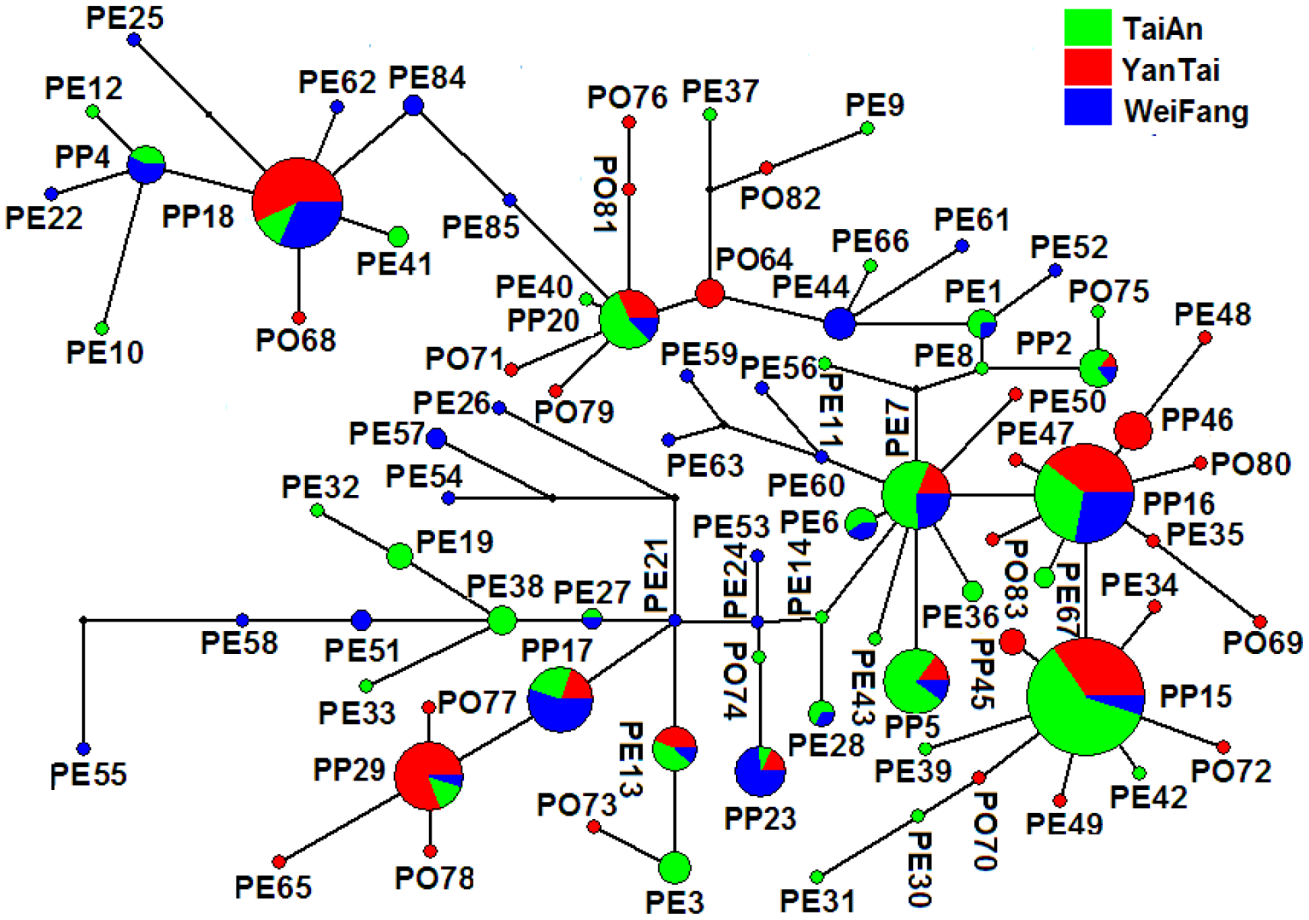
Median-Joining network based on the combination of *G. molesta* COI and COII mtDNA haplotypes. Each circle represents a haplotype, and the area of a circle or square is proportional to the number of observed individuals. Colors within the nodes refer to the *G. molesta* sampling regions: green, TaiAn; red, YanTai; blue, WeiFang; black, the inferred missing haplotypes. The two capital letters each, PE, PO and PP indicate haplotpyes from peach, pome fruit (apple and pear) and both peach and pome fruit, respectively.

A total of 85 haplotypes were obtained with the combination of COI and COII genes. The network of the haplotypes are shown in Fig. 4. The nine most common haplotypes were shared by the populations of all the three regions (YanTai, TaiAn and WeiFang), of which eight were obtained from both peach and pome fruits (PP5, PP15, PP16, PP17, PP18, PP20, PP23 and PP29), one from peach (PE7). In TaiAn, WeiFang and YanTai, 40, 37 and 35 haplotypes were observed, respectively. Populations from all the three regions showed a high number of private haplotypes (with 24, 21 and 24 in TaiAn, WeiFang and YanTai, respectively). TaiAn and WeiFang populations shared five haplotypes (PE1, PE6, PE27, PE28 and PP4). No other haplotypes were observed to be shared by two regions.

#### NJ tree of the combined COI and COII haplotypes

**Figure 5:**
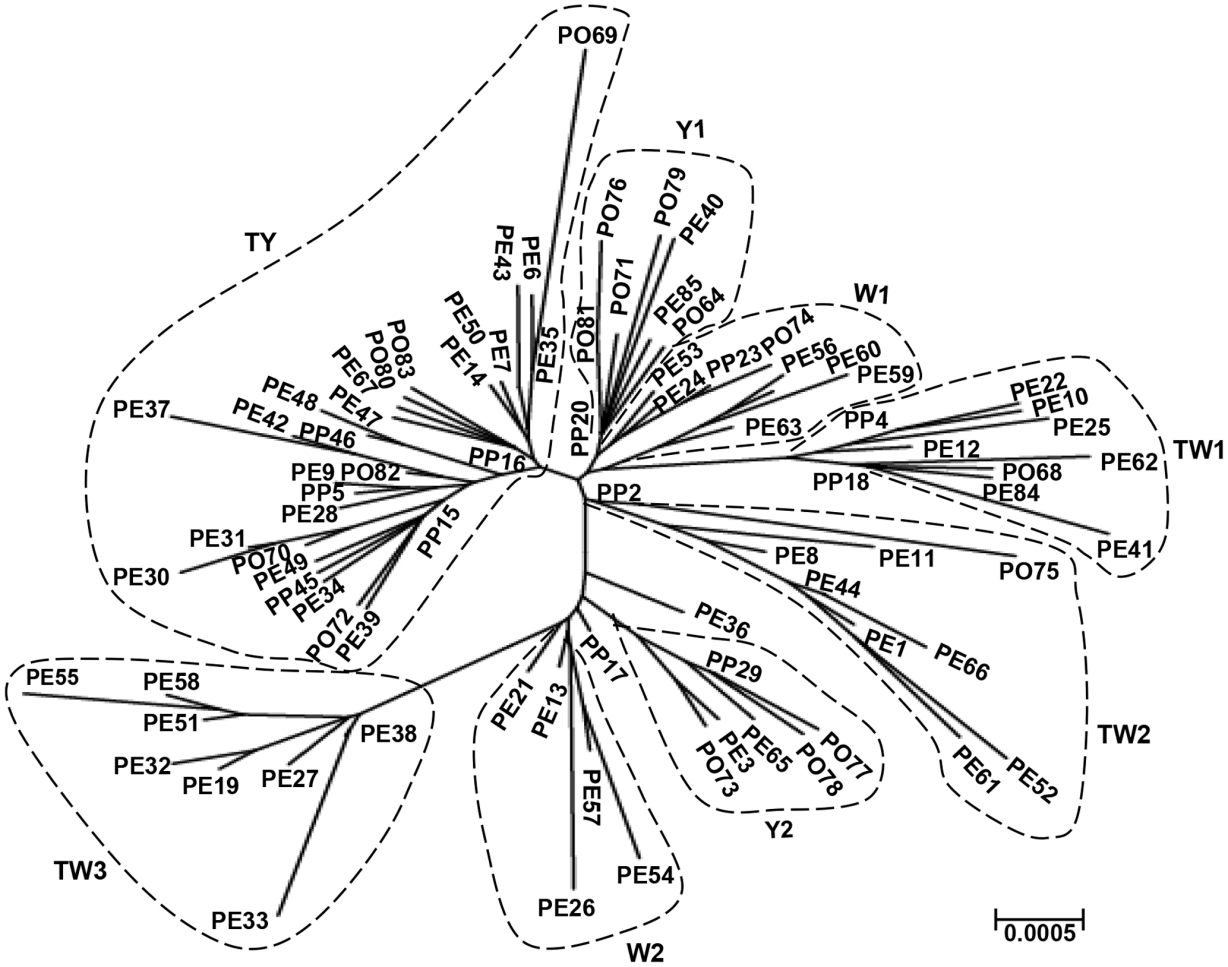
Unrooted NJ phenograms based on the combination of *G. molesta* COI and COII mtDNA haplotypes. Eight clusters are indicated by TY, TW1,TW2, TW3, W1, W2, Y1 and Y2.

NJ tree of combined *G. molesta* COI and COII haplotypes based on p-distance is shown in Fig. 5. The analysis based on the combination of COI and COII gene divided the haplotypes into eight major clusters (W1, W2, Y1, Y2, TW1, TW2, TW3 and TY). The W1 and W2 cluster mainly contained the haplotypes identified in the WeiFang region. The Y1 and Y2 cluster mainly comprised haplotypes from the YanTai region. The TW1, TW2 and TW3 cluster was primarily a mixture of haplotypes from both the TaiAn and WeiFang region. The TY cluster was mainly made up of haplotypes from both the TaiAn and YanTai region.

### Analysis of molecular variance (AMOVA)

AMOVA results for the three models are shown in Table 5. Significant, albeit low level genetic variances were found in all three models, with populations grouped according to (A, B) region and (C) type of host plants.

**Table 5:** AMOVA results of microsatellite data comparing genetic variation in *G. molesta* populations using three models.

Model	Populations	Source of variation	d.f.	Sum of squares	Variance components	Percentage of variation	*P* value
A	Populations from peach in three regions	Among regions	2	63.43	0.118	5.63	*P*<0.001
		Among populations within regions	10	99.62	0.17	8.18	*P*<0.001
		Within populations	609	1096.05	1.78	86.17	*P*<0.001
B	Populations from pome fruit (apple and pear) in three regions	Among regions	1	5.44	0.02	1.02	*P*<0.05
		Among populations within regions	2	14.60	0.13	7.67	*P*<0.001
		Within populations	172	275.65	1.60	91.31	*P*<0.001
C	Populations from different types of host plants (peach and pome fruit)	Among different types of host plants populations	1	19.58	0.03	1.33	*P*<0.001
		Among individuals within populations	15	185.25	0.22	10.70	*P*<0.001
		Within individuals	781	1435.73	1.84	87.97	*P*<0.001

(A) populations collected from peach of three regions; (B) populations collected from pome fruit (apple and pear) of different regions; (C) populations collected from different types of host plants (peach and pome fruit).

## Discussion

We investigated the genetic diversity and genetic structure of 17 *G. molesta* populations from three regions of a main fruit growing area in China, using microsatellite and mitochondrial DNA markers. High genetic diversity was documented in these moth populations. All populations from peach were geographically structured. Whereas genetic structure of populations from peach was similar throughout the growing season within each region, populations collected from apple and pear in the late season surprisingly differed in genetic structure from populations collected from peach.

### Genetic diversity

This study provided an extended sample analysis from the putative native region of *G. molesta*. The mean number of alleles per locus (*N_A_*) averaged 10.4 over all populations, which was about twice as high as reported for *G. molesta* populations that invaded Italy (average 5.8) [24], Australia, South Africa, South and North American and European countries (average 4.4) [3]. Moreover, we found high numbers of private alleles of microsatellite loci and high allelic richness at each sublocation sampled. All these characteristics of high genetic diversity found with microsatellites are indicators of a native species [48]–[50]. Furthermore, all populations displayed large numbers of mitochondrial haplotypes, with a total of 86 haplotypes obtained for COI (average 5.1 over populations), and of 138 haplotypes for COII (average 8.1 over populations). High numbers of private haplotypes existed in each of the three regions. Both these characteristics found with mitochondrial markers are again indicators of native species, especially for native species with low dispersal capacity [7], [50]. In fact, the criterion of low dispersal capacity applies also to *G. molesta*, which has the flight ability to move between non-contiguous orchards but not over long distances [17], [23], [51]. Previous molecular studies on the oriental fruit moth relied on populations sampled globally or in invaded regions [3], [24], whereas our data provide for the first time comprehensive empirical evidence for the assumption [9] that China lies indeed in the range of *G. molesta*'s origin.

### Geographical genetic structure

Populations from peach were geographically structured in the three sampled regions of China, as documented by the well defined clusters formed by populations from each region based on microsatellite markers. There was weak but significant correlation between geographical distance and genetic distance among these populations. Our microsatellite data provided stronger information on geographical population structure than mitochondrial data did in the NJ tree of the combination of COI and COII haplotypes. This difference may be due to the diploid nature of microsatellites as opposed the haploid nature of mitochondrial markers, as was concluded in a review on population genetics information obtained from these markers [7]. The sampling in the present study was carried out in one of the most important fruit growing areas of China with a long history of fruit tree planting. The geographically structured populations found are likely the result of the limited flight capacity of *G. molesta* [17], [23], [51], so that isolation sufficed to restrict spread of the species. Our findings from China are in line with results from South Africa on localized populations [52], from Italy on significant intra-regional structure [24], and from a global survey reporting geographical structure on a continental scale [3]. Similar results on genetic structure were found in a closely related moth species with low flight capacity, *Cydia pomonella* [5], [52]–[54]. In the current study, we found consistently similar genetic structures within a region (Fig. 2), and human-mediated displacement [55] might have contributed to this result. Actually, local Chinese governments promote centralized purchase of fruit, what might facilitate genetic exchange of *G. molesta* populations within regions, as is also the case for cooperatives in Italy [24].

Genetic variance among all populations sampled existed (Table 5). The only exception (6TTY-Ph and 7TYT-Ph) without significant genetic variance (*P* = 0.216) was in a heavily infested abandoned orchard, which seemed to have provided a particularly stable environment for the moth. Otherwise, environmental conditions in single orchards sufficiently changed between sampling times to allow for detection of significant genetic variance. Such environmental changes include the considerable changes in emitted volatile blends from host trees with progressing season, which elicit altered behavioral responses of the oriental fruit moth [18], [20].

### Genetic structure across host plants

Populations sampled in the late season from the pome fruit apple and pear differed in genetic structure from populations sampled from the primary host peach, and they form a single clade exclusively comprising populations from pome fruit. Three factors might have contributed to this remarkable finding: the increased flight performance of the moth in the late season [56], the altered olfactory preference of gravid female to changing volatile blends emitted by peach and pome fruit with progressing season [18]–[20], and the recently detected existence of different olfactory genotypes in this moth species [21]. The first two factors are associated with the movement of the moths from the stone fruit peach to the pome fruit apple and pear. Decreasing length of daylight experienced by the growing larvae promote enhanced flight performance of the emerged *G. molesta* moths, and this effect is even stronger when ambient temperature is still high [56]. Furthermore, the volatiles emitted by apple and pear become attractive for the female moths towards the late season, facilitating a host switch during this period [18], [20]. Beyond these two factors, which increase flight and facilitate orientation towards pome fruit trees, the following third factor may contribute to explain the different genetic structure of the populations from pome fruit compared to populations from peach in the late season. In the oligophagous *G. molesta*, there are different olfactory genotypes, and this natural genetic diversity facilitates host switch [21] and likely contributes to the differential population structure documented in the current study for larvae from pome fruit compared to those from peach. Our results from the population structure analysis and from the NJ tree consistently suggest a selective host switch of a certain part of the population only. Certain genotypes from peach, possibly those particularly responsive to pome fruit odors, seem to be the major contributors to the host switch.

### Implication for pest management

The oriental fruit moth is not only a key pest of rosaceae fruit trees in its native range in Asia, requiring major pest management input [11], [12], [57], but has invaded all other fruit growing continents, posing major challenges to sustainable fruit tree protection [10], [51], [58]. The current study is the first to show that population genetic structure of this pest may be changing with the host switch from the primary host peach to the secondary hosts pear and apple in the late part of the season, and this finding might provide useful information for the design of future season-long pest management programs. For example, late season pest management in pome fruit might rely on different pest management tools than those used in the early season on peach. This strategy holds the potential for two benefits: first, it can be adjusted optionally to the genotypes prevailing in the respective pest populations, and second, the alternation of the management approach in the second part of the season might contribute to limit univocal selection pressure and thus forced evolution of resistant pest genotypes [20], [59], [60].

## Conclusions

Genetic diversity of *G. molesta* is high in its region of origin. Significant genetic structure exists among populations from peach in the different regions. The weak flight capacity of the moth underlies the pattern of genetic structure observed. Contrary to our original expectation, host switch of the moth results in different genetic structure of populations on the secondary host plants, to which the recently detected different olfactory genotypes of the moth likely contribute. Design and implementation of sustainable pest management strategies could therefore be adjusted to the population genetics and dispersal of this pest insect.
